# Hypernatremia in brain‐dead patients

**DOI:** 10.1002/brb3.2574

**Published:** 2022-04-22

**Authors:** Weixin Guo, Shouhong Wang, Zhonghua Wang, Peihang Hu, Xuebiao Wei, Xiaolong Liao

**Affiliations:** ^1^ Guangdong provincial Geriatric's Institute Guangdong Provincial People's Hospital Guangdong Academy of Medical Sciences Guangzhou China

**Keywords:** brain death, brain natriuretic peptide, coma, hyponatremia, intensive care

## Abstract

**Objectives:**

Hypernatremia often occurs in patients with brain death. This study summarizes its characteristics.

**Methods:**

We recorded 57 patient's highest blood sodium value, as well as daily NT‐proBNP, blood creatinine, and urine output. Further, we analyzed the time of the first rise in blood sodium, and the relationship between NT‐proBNP, serum creatinine, urine output, and serum sodium.

**Results:**

There was no hyponatremia in these patients, and only seven of the 53 patients registered blood sodium between 137 and 150 mmol/L. We found that blood sodium started to rise at 36.0 (28.5–52.3) h, reaching the highest value in 79.0 (54.0–126.0) h. Urine volume and creatinine have no correlation with serum sodium level, while NT‐proBNP has a significant correlation with serum sodium level.

**Conclusion:**

It is necessary to conduct volume assessments and urine electrolyte testing on patients with brain death. BNP has a protective effect on water and electrolytes to prevent hypernatremia.

## Graphical abstract

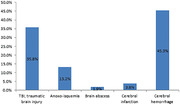

## 1 INTRODUCTION

Hypernatremia is a common issue encountered during the pretransplant care of brain‐dead organ donors (Aiyagari et al., 2006). Hypernatremia can cause treatment difficulty for the clinic as it cares for brain‐dead patients. Not only does hypernatremia play an important role in hemodynamic stability, but it may also influence organ transplantation outcomes. Hypernatremia is common among patients with neurological symptoms; it is also a risk factor for mortality (Noda & Hiyama, 2015). In this situation, substitution with common electrolyte solutions can induce disturbances of water and electrolyte balance (edema, hyperosmolarity), particularly in terms of cell membrane and organ function deterioration. Hypernatremia associated with hypertonicity determines the passage of water from inside the cell to the interstitium, causing cellular dehydration as well as a decrease in cell volume (Imaizumi et al., 2017). It is not uncommon for organ donors to have elevated levels of plasma sodium and osmolarity, which can sometimes be difficult to normalize despite the use of intravenous hypo‐osmolar sera. In our literature review, we found that the occurrence of hypernatremia in patients with brain death may be related to urine output, NT‐proBNP, renal function, and the use of mannitol. This study aims to discover some of the regularities of hypernatremia in patients with brain death. Such regularities were examined through the cases accumulated in our hospital for 4 years, including the proportion of hypernatremia in patients with brain death, when hypernatremia increases in brain‐dead patients, whether hypernatremia is related to the urine output and the NT‐proBNP, and whether the blood creatinine and use of mannitol were related. We hope that our research can effectively prevent the occurrence of hypernatremia and contribute to the protection of donor organs.

## 2 MATERIALS AND METHODS

Descriptive, retrospective, and observational studies were carried out. The study was carried out over an extended period from December 2017 to January 2021. A total of 53 brain‐dead patients were collected in this study, and all patients met “the criteria and practical guidance for determination of brain death in adults (2nd edition).” All patients were organ donors. All patients underwent electroencephalogram (EEG), evoked potentials, transcranial Doppler (TCD), and breath provocation tests. We recorded each patient's highest blood sodium value, as well as each day's NT‐proBNP, blood creatinine, and urine output (Table 1). We analyzed the time when blood sodium first rose, the relationship between NT‐proBNP and serum sodium, the relationship between serum creatinine and serum sodium, and the relationship between urine output and serum sodium.

**TABLE 1:** Total volume of fluid intake and output within 12 h

	N	Min (ml)	Max (ml)	Mean	SD
Intake	31	818	4389	2053.42	778.07
Urine output	31	500	3235	1958.32	685.50
Total output	31	510	3800	2057.74	766.23

Statistical analyses were performed using the SPSS software (Statistical Package for the Social Sciences, version 17.0, SPSS Inc, Chicago,Ill, USA). Correlations of quantitative variables were analyzed by Spearman rho correlation tests. Differences between the groups were assessed by the *t* test. *p*‐Value of less than 0.05 was considered significant. The study was approved by the research ethics committee of Guangdong Provincial People's Hospital (2019‐792H). Written informed consent was obtained from all included patents’ family.

## 3 RESULTS

### 3.1 Frequency distribution of pathologies

**FIGURE 1:**
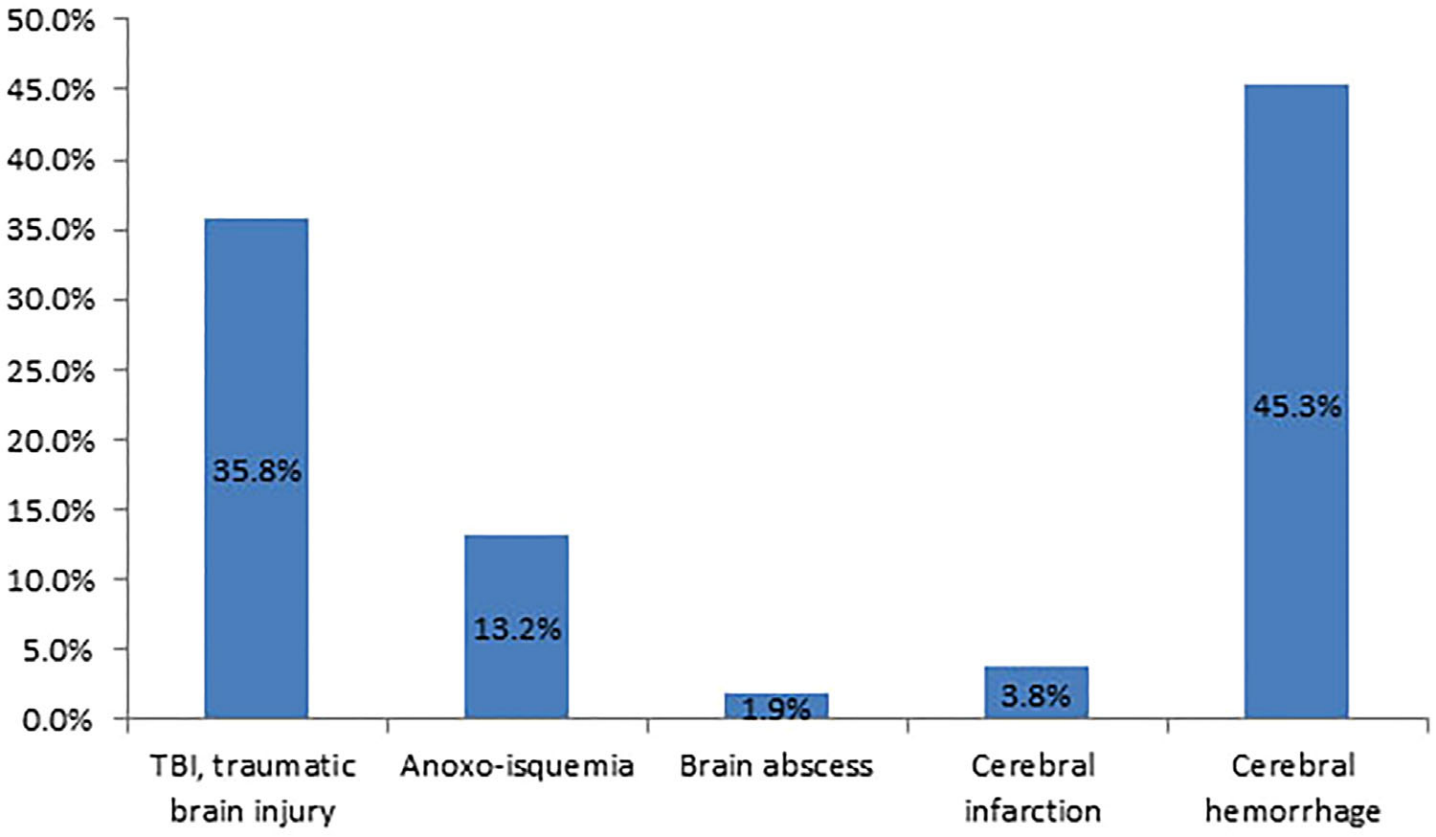
Frequency distribution of pathologies

During the period considered, 53 patients were surveyed, who were admitted to intensive care units or the Emergency Department in our hospital. Distribution by sex was 75% male and 25% female, and the *ẋ* of age was 41.92 ± 13.64 years old. The age range was from 14 to 66 years (Figure 1) (Table 2).

**TABLE 2:** The frequency distribution of pathologies corresponded to the following

Pathologies	Number of patients	Frequency
TBI, traumatic brain injury	19	35.8%
Anoxo‐isquemia	7	13.2%
Brain abscess	1	1.9%
Cerebral infarction	2	3.8%
Cerebral hemorrhage	24	45.3%
Hypertensive cerebral hemorrhage	10	18.9%
Aneurysm	7	13.2%
Arteriovenous malformations	1	1.9%
Cerebrovascular surgery	2	3.8%
unknown reason	4	7.5%

### 3.2 Time to elevated blood sodium

**FIGURE 2:**
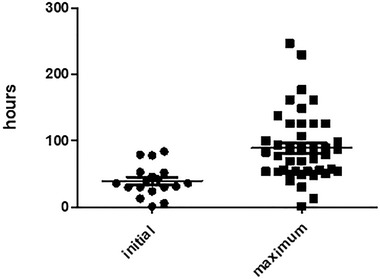
Time to elevated blood sodium

The days when blood sodium started to rise and when blood sodium reached the maximum were recorded. Because some patients were transferred from an outside hospital, we could not record the day when the increase first started, and we removed patients without elevated sodium. When the highest blood sodium was recorded, the absence of elevated blood sodium and renal replacement therapy were removed. Eighteen patients were included in the statistics in the initial ground 36.0 (28.5–52.3) h. Forty‐three patients were included in the statistics in the maximum ground 79.0 (54.0–126.0) h (Figure 2).

### 3.3 Correlation analysis of Na and NT‐proBNP

**FIGURE 3:**
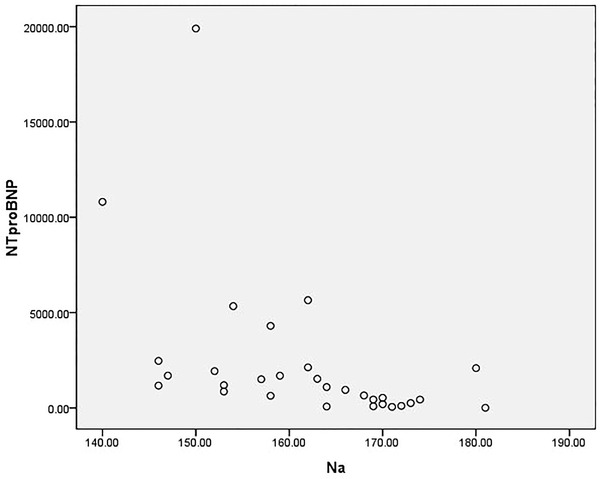
Correlation analysis of Na and NT‐proBNP

Selected patients without renal replacement therapy were tested for blood sodium levels and had blood drawn to test brain natriuretic peptide precursor. A total of 32 patients were included in this calculation. The values of serum sodium ranged (140.0–183.0) mmol/L, and the median (interquartile) value was 163.5 (153.3–170.0) mmol/L. The lowest value of NT‐proBNP was 5 pg/ml, and the highest value was 19,903 pg/ml, the median (interquartile) of NT‐proBNP was 1057.5 (298.3–2047.8) pg/ml. Their distribution was as follows: the abscissa was the blood sodium concentration (mmol/L), and the ordinate was the NT‐proBNP concentration (pg/ml). Spearmen correlation analysis showed that there was a significant correlation between high serum sodium levels and NT‐proBNP, and the difference was statistically significant (*r*s = −0.671, *p* < .001), thereby suggesting that the patient's NT‐proBNP level decreased as the patient's serum sodium increased (Figure 3).

### 3.4 Correlation analysis of Na and urine volume

**FIGURE 4:**
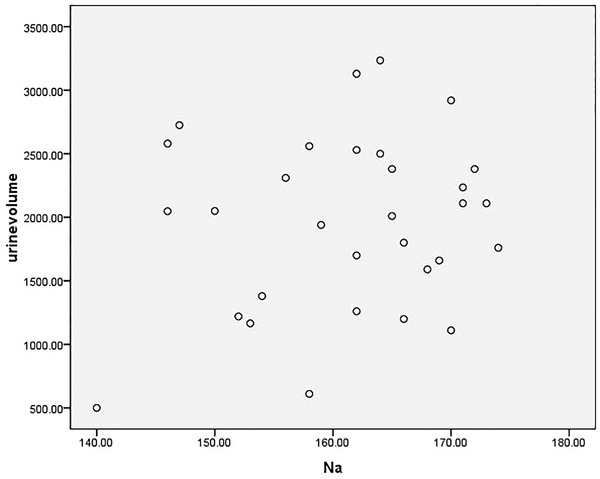
Correlation analysis of Na and urine volume

Select patients without renal replacement therapy were tested for blood sodium levels. Such patients had blood drawn time as the time point to Calculate 12 hours urine output before this time. A total of 31 patients were included in this calculation. The lowest value of serum sodium is 140 mmol/L, the highest value is 174 mmol/L the mean value of serum sodium is (161.13 ± 9.06) mmol/L; the minimal urine output is 500 m/12 h, and the maximum urine output is 3235 ml/12 h [2048.0(1380.0–2500.0) ml/24 h]. Their distribution was as follows: the abscissa was the blood sodium concentration (mmol/L), the ordinate was the urine output (ml/12 h). Spearmen correlation analysis showed that there was no significant correlation between high serum sodium levels and urine output, and the difference was not statistically significant (rs = 0.076, *p* = .683) (Figure 4).

### 3.5 Correlation analysis of Na and blood creatinine

**FIGURE 5:**
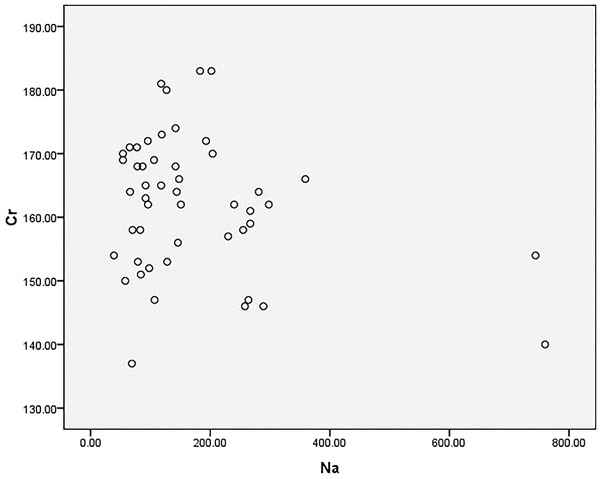
Correlation analysis of Na and blood creatinine

Select patients without renal replacement therapy, were tested for blood sodium levels and had blood drawn to test creatinine. A total of 49 patients were included in this calculation. The lowest value of serum sodium was 137 mmol/L, the highest value was 183 mmol/L, the mean value is 162.12 ± 10.64 mmol/L; the lowest value of creatinine was 39 pg/ml, and the highest value was 760 pg/ml 127.0 (83.5–235.0) pg/ml. Their distribution was as follows: the abscissa was the blood sodium concentration (mmol/L), the ordinate was the creatinine (umol/ml). Spearman correlation analysis showed that there was no correlation between high serum sodium levels and creatinine (rs = −0.13, *p* = .385) (Figure 5).

### 3.6 Comparison of serum sodium in the two groups with and without mannitol

**FIGURE 6:**
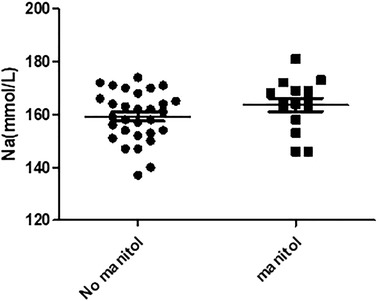
Comparison of serum sodium in the two groups with and without mannitol

In this analysis, there were a total of 45 patients without renal replacement therapy, 30 in the mannitol group (159.20 ± 9.47 mmol/L), and 15 in the nonmannitol group (163.60 ± 9.69 mmol/L). To record the highest blood sodium value while recording the blood creatinine value, we performed an independent sample *t* test. The *t* value was −1.46 (*p* = .152). There was no statistical difference between the two groups (Figure 6).

## 4 Discussion

The important goal in the care of brain‐dead potential organ donors is to stabilize their hemodynamic status (Aiyagari et al., 2006). Na+ homeostasis is associated with body fluid control. Critically ill patients with neurological diseases are prone to developing hypernatremia (Noda and Hiyama, 2015), and it has been reported that hypernatremia increases the mortality rate among critically ill patients with cerebrovascular diseases (Imaizumi et al., 2017). This study collected data from brain‐dead patients in our hospital from December 2017 to January 2021. Among these patients, there was no hyponatremia, and only seven out of 53 patients had blood sodium between 137 and 150 mmol/L.

Hypernatremia should be corrected as soon as possible to make a clinical diagnosis of BD, and to avoid its potentially harmful effects on subsequent organ function support. Here, we summarize the data collected from these patients. In as soon as 36.0 (28.5–52.3) h, we found that blood sodium started to rise, reaching the highest value in 79.0 (54.0–126.0) h. Hypernatremia in patients with brain death mainly leads to internal environmental disorders that can lead to organ dysfunction, which presents a challenge in the protection of donor organs (Kutsogiannis et al., 2006). From our results, we found that when blood sodium is greater than 145 mmol/L, the blood sodium may reach its peak in 79 h. We hope in the future that we can consciously prevent it as soon as possible.

It is difficult to correct hypernatremia in patients with brain death, just as it is difficult to correct hypernatremia even without the use of sodium solvents when strengthening diuresis. It often requires renal replacement therapy to reduce sodium (Hemphill et al., 2015, Toyoda et al., 2013). Undoubtedly, it increases the difficulty of organ maintenance as well as medical expenses. The cause of hypernatremia in patients with brain death is what we hope to uncover through this study. Because of the completeness of the data, we collected and analyzed the data from NT‐proBNP, urine output, blood creatinine, and mannitol treatment.

The results showed that there was no correlation between urine output and blood sodium levels. Whether or not mannitol treatment is used had no effect on blood sodium levels. NT‐proBNP and serum creatinine are negatively correlated with hypernatremia. These results present a conflict: it is reasonable to expect urine output to be related to blood sodium, but after repeatedly checking, our urine output is decidedly calculated as the 12‐h urine output before the blood sodium test. The scatter plot indicates that the data follows no predictable pattern. We pose the following analyses: the first possibility is that the patient's capacity is involved. Most patients suffer from diabetes insipidus in a brain‐dead state, and the capacity is difficult to detect. Almost 74% of the donors in our series had polyuria (urine output greater than 125 ml/h; Ramos and Lopez, 2002, Hirschl et al., 1996, Seyed Mohammad et al., 2008).

For example, if the patient has insufficient capacity, the amount of sodium taken away in the urine output is directly related to the concentration of blood sodium. In diabetes insipidus, the amount of urine does not indicate the amount of capacity. It can be detected by B‐ultrasound or pulse index continuous cardiac output (PICCO). Our data only considers urine output. In this study, the urine output of patients is generally too large.

Second, there is no correlation between our serum creatinine and serum sodium. The possible reason is that the patients in this study generally have large urine volumes and the original renal function is normal. Patients with brain death are likely to experience acute kidney injury due to insufficient capacity. In acute kidney injury, renal tubular function is impaired, and hyponatremia is caused by decreased sodium retention capacity. Studies have shown that in acute renal ischemia, the expression of sodium transporter in the proximal tubules and distal tubules of the kidney decreases during perfusion, which leads to the decline of the renal sodium retention capacity (Wang et al., 1998). The above is our hypothesis and needs future research. Based on the above point, it is necessary to conduct volume assessments and urine electrolyte testing on patients with brain death.

The results suggest that there is no difference in blood sodium after the use of mannitol. The idea that mannitol causes blood sodium to increase has been controversial. There is a clinical study in which the data indicates that mannitol can cause both hypernatremia and hyponatremia, and the research object is patients with severe brain injures (Seo & Oh, 2010). In animal experiments, it has been clearly indicated that mannitol is not related to hypernatremia. This study believes that we have sodium ions regulating cells in the brain, and that these [Na+]‐sensitive cells were insensitive to the rise in osmolality or [Cl‐], but sensitive to serum sodium. We hypothesize that these brain cells are in an apoptotic state in patients with brain death, leading to sodium regulation disorders, and hypernatremia in patients with brain death. Due to the limited number of patients involved in our study, we hope to observe more cases in the future (Hiyama et al., 2010; Hall, 2004).

Brain natriuretic peptide (BNP) is a 32 amino acid cardiac natriuretic peptide hormone originally isolated from porcine brain tissue. NT‐proBNP (76 amino acids) can be measured by immunoassay in human blood. Under the action of proBNP endodicerase, it is cleaved into BNP with beneficial sodium, diuretic, vasodilator, and other biological activities, and nonbiologically active N‐terminal pro‐B‐type natriuretic peptide (NT‐proBNP) (Maisel et al., 2002). Measurements of BNP and the amino terminal portion of the pro‐hormone (NT‐proBNP) are already available for clinical use in many European countries and in the United States. European health authorities have approved BNP and NT‐proBNP testing to assist clinicians in the diagnosis of heart failure (HF) (Bettencourt et al., 2000; Koenig et al., 2007). Both peptides are released into the blood circulation. Both have the same source and are secreted equimolar. Therefore, theoretically, the clinical application results of detecting BNP and NT‐proBNP are the same, and there is not much difference in terms of years of clinical results (Levin et al., 1998). They are quantitatively different due to the disparity between half‐life and metabolic pathways, but they will have a consistent correlation, and in our study, NT‐proBNP was detected.

Natriuretic peptides are molecules that normally defend against periods of excess water and salt retention by antagonizing the RAAS system, promoting vascular relaxation, and inhibiting excess sympathetic outflow and the generation of vasoconstrictor peptides (Yee et al., 2010, Isotani et al., 1994). Some investigators have proposed that the generation and release of natriuretic peptides from the hypothalamus in disease states, such as SAH, may serve a protective role against elevated intracranial pressure. Direct damage to cortical and subcortical structures where BNP exists leads to inadvertent release of hormones directly into blood circulation (Isotani et al., 1994). BNP can reduce the pre‐ and postload of the heart and increase renal perfusion. Through this study, we found that BNP also has a protective effect on water and electrolytes to prevent hypernatremia. Whether it is present in patients with severe brain injury and brain death is also a topic worthy of further study.

## FUNDING INFORMATION

None.

## CONFLICT OF INTEREST

The authors declare no conflict of interest.

## AUTHOR CONTRIBUTIONS

WX‐G is responsible for the experiment and drafting the manuscript. XL‐L assisted in the experiment. ZH‐W assists in conceiving and drafting manuscripts. PH‐H helps to analyze experimental data. XB‐W assisted in completing the manuscript. SH‐W helped to revise the manuscript. Everyone has read and agreed to the final manuscript.

### PEER REVIEW

The peer review history for this article is available at https://publons.com/publon/10.1002/brb3.2574.

## Data Availability

Data sharing not applicable to this article as no datasets were generated or analysed during the current study.
